# Bee Product-Based Antimicrobial Film-Forming Gels Targeting *Staphylococcus aureus*, *Staphylococcus epidermidis*, and *Cutibacterium acnes* for Anti-Acne Applications

**DOI:** 10.3390/gels11100802

**Published:** 2025-10-06

**Authors:** Suvimol Somwongin, Pattiya Tammasorn, Ratthaporn Limbunjerd, Kankamon Norkaew, Nattakan Lertprachyakorn, Thanaphorn Kongsaeng, Patcharin Phokasem, Terd Disayathanoowat, Wei-Chao Lin, Wantida Chaiyana

**Affiliations:** 1 Department of Pharmaceutical Sciences, Faculty of Pharmacy, Chiang Mai University, Chiang Mai 50200, Thailand; suvimol.s@cmu.ac.th (S.S.); pattiya_tammasorn@cmu.ac.th (P.T.); ratthaporn_lim@cmu.ac.th (R.L.); kankamon_nor@cmu.ac.th (K.N.); 2 Research Center of Deep Technology in Beekeeping and Bee Products for Sustainable Development Goals: SMART BEE SDGs, Chiang Mai University, Chiang Mai 50200, Thailand; patcharin.ph@cmu.ac.th (P.P.); terd.dis@cmu.ac.th (T.D.); 3 School of Cosmetic Science, Mae Fah Luang University, Chiang Rai 57100, Thailand; 6431701115@lamduan.mfu.ac.th (N.L.); 6431701126@lamduan.mfu.ac.th (T.K.); 4 Office of Research Administration, Chiang Mai University, Chiang Mai 50200, Thailand; 5 Department of Biology, Faculty of Science, Chiang Mai University, Chiang Mai 50200, Thailand; 6 Department of Cosmetic Science and Institute of Cosmetic Science, Chia Nan University of Pharmacy and Science, Tainan 71710, Taiwan; weilin@mail.cnu.edu.tw; 7 Multidisciplinary and Interdisciplinary School, Chiang Mai University, Chiang Mai 50200, Thailand; 8 Center of Excellence in Pharmaceutical Nanotechnology, Faculty of Pharmacy, Chiang Mai University, Chiang Mai 50200, Thailand

**Keywords:** propolis, honey, royal jelly, anti-acne, film-forming gel

## Abstract

This study aimed to develop an optimized film-forming gel for topical anti-acne applications by evaluating the antibacterial efficacy of propolis, honey, and royal jelly, individually and in combination with low-dose salicylic acid. The antibacterial activities were assessed against *Staphylococcus aureus*, *Staphylococcus epidermidis*, and *Cutibacterium acnes* using the inhibition zone assay. Film-forming gels were developed by evaluating the effects of type and concentration of polymers and plasticizers. Each formulation was evaluated for visual appearance, pH, viscosity, and drying time, along with the appearance of the corresponding film. The findings noted that propolis (1% *w*/*w*) exhibited the strongest antibacterial activity among individual bee products, producing an inhibition zone of 20.0 ± 1.0 mm against *S. aureus*. The combination of bee products with low-dose salicylic acid (0.1% *w*/*w*) markedly enhanced antibacterial efficacy, particularly against *C. acnes* (inhibition zone 40.8 ± 0.8 mm). Incorporation of this combination into the optimized film-forming gel, containing polyvinyl alcohol, Carbomer^®^ 940, polyethylene glycol 400, glycerin, and water, produced a formulation with balanced pH, suitable viscosity, 31 min drying time, and complete inhibition of *S. aureus* and *S. epidermidis*. Therefore, bee product-based film-forming gels, combined with low-dose salicylic acid, exhibited favorable physicochemical properties and showed promise as complementary anti-acne therapies.

## 1. Introduction

Acne vulgaris is a common chronic inflammatory skin disorder that affects millions of individuals worldwide, most frequently adolescents and young adults, though it can occur at any age and often persists into adulthood [1]. The condition is characterized by the development of comedones, papules, pustules, and nodules, primarily due to the hyperkeratinization of follicles, excessive sebum production, colonization by acne-associated bacteria, and inflammatory responses [2,3]. Among the microbial contributors, *Cutibacterium acnes* is recognized as the primary pathogen, with *Staphylococcus aureus* and *Staphylococcus epidermidis* also implicated in the inflammatory cascade [4,5]. Standard topical treatments include salicylic acid, benzoyl peroxide, and antibiotics such as tetracycline and gentamicin [6,7]. However, their prolonged or high-concentration use can result in adverse effects such as skin irritation, dryness, erythema, peeling, and, in rare cases, systemic toxicity, leading to limited patient compliance [7,8]. Consequently, there is growing interest in natural bioactive compounds that can complement or reduce the reliance on conventional chemical agents while providing antimicrobial and skin-beneficial effects.

Bee-derived products, including propolis, honey, and royal jelly, are rich sources of bioactive compounds such as flavonoids, phenolic acids, peptides, and proteins [8,9,10]. Propolis has been widely reported for its potent antibacterial, anti-inflammatory, and antioxidant properties, demonstrating efficacy against a variety of skin pathogens, including *S. aureus* and *C. acnes* [11]. A topical solution of ethanolic propolis extract demonstrated clinical efficacy in acne vulgaris with significant activity against *S. epidermidis* and *C. acnes* [12]. Additionally, propolis extract has been reported to be effective against acne by disrupting *C. acnes* biofilms and modulating the skin microbiota [13]. The efficacy of 20 mg/mL propolis has been reported to be greater than that of 10% benzoyl peroxide in the *C. acnes* inhibition [14]. Honey exhibited high sugar content and hygroscopic properties, contributing to wound healing, hydration, and antibacterial effects [15,16], whereas royal jelly contained proteins and vitamins that exerted protective effects on reproductive health, neurodegenerative disorders, wound healing, and aging [17]. Honey alone has been reported to inhibit *S. epidermidis* and *C. acnes* with minimal inhibitory concentrations of 50% *v*/*v* for both bacteria, but enhanced efficacy was observed when combined with other natural extracts [18]. Additionally, royal jelly has been reported for strong antibacterial activity against *S. aureus* and *C. acnes*, along with anti-inflammatory effects by reducing nitric oxide production and suppressing inducible nitric oxide synthase (iNOS) expression [19]. These previous studies highlighted the potential of various bee-derived products as alternative therapies for acne. The strategic combination of these natural products, along with low concentrations of conventional agents such as salicylic acid, may enhance antibacterial efficacy, reduce the required dose of synthetic chemicals, and minimize potential side effects, offering a promising approach for acne management.

Nowadays, various topical treatments for acne vulgaris are available, including creams, gels, solutions, ointments, and patches [20]. Novel formulations, such as film forming systems, which are non-solid dosage forms that create a film in situ upon application, provided an effective alternative to conventional topical and transdermal treatments [21]. Topical delivery via film-forming gels offers distinct advantages for anti-acne therapy, combining the benefits of conventional gels with the ability to form a thin, uniform film on the skin. Previous study reported that α-Mangostin-loaded film-forming gels demonstrated strong antibacterial activity against *S. aureus* and *C. acnes* while maintaining good physicochemical stability during storage [20]. This film forming system would ensure uniform application, prolonged contact, and controlled release of active agents, including bee-derived products and low-dose salicylic acid, enhancing therapeutic efficacy while minimizing the required concentration of chemical agents and reducing the risk of irritation or other adverse effects [22]. Moreover, the resulting film is transparent, flexible, and non-greasy, improving cosmetic acceptability and patient compliance, critical factors in managing chronic conditions like acne. The film also provides a protective barrier that maintains skin hydration and shields the affected area from external contaminants, further supporting skin healing and comfort [23]. Together, these properties make film-forming gels an effective, patient-friendly delivery platform and a rational choice for developing multi-component, natural product-based anti-acne formulations.

Therefore, the present study aimed to develop film-forming gels as novel anti-acne formulations designed to overcome the limitations of conventional topical treatments. By combining bee-derived products with low-dose salicylic acid, the formulation was expected to reduce the chemical dose while maintaining or enhancing therapeutic efficacy. In addition to its antibacterial activity, this approach might minimize the risk of skin irritation or other adverse effects, while providing additional skin benefits from the antioxidant, anti-inflammatory, and moisturizing properties of bee-derived products [15,16,17]. This study focused on evaluating the antibacterial efficacy of propolis, honey, and royal jelly, both individually and in combination with low-dose salicylic acid, and on developing an optimized film-forming gel formulation with balanced physicochemical properties, suitable for potential topical anti-acne applications.

## 2. Results and Discussion

### 2.1. Antimicrobial Properties of Bee Products

The antibacterial activities of individual bee products, their combinations, and reference compounds were evaluated against acne-related bacteria, including *S. aureus*, *S. epidermidis*, and *C. acnes*, using the inhibition zone assay, as shown in Table 1. Among the reference antibiotics, tetracycline exhibited greater efficacy than gentamicin at the same concentration, except against *S. aureus*, where their effects were comparable. In addition, salicylic acid at a higher concentration of 0.35% *w*/*v* demonstrated strong antibacterial activity, comparable to that of 0.1% *w*/*v* tetracycline. However, salicylic acid is commonly found in over-the-counter acne products at concentrations of 2% *w*/*w* or higher and is also included in some prescription formulations [7]. In certain cases, a 1.5% *w*/*w* salicylic acid cream, combined with natural skin penetration enhancers and antioxidants, has been used to treat facial acne [24].

Interestingly, various bee products also presented promising anti-acne efficacy. Among the bee products tested, propolis extract (1% *w*/*w*) exhibited the strongest antibacterial activity, producing an inhibition zone of 20.0 ± 1.0 mm against *S. aureus*, which was comparable to gentamicin (0.1 mg/mL), tetracycline (0.1 mg/mL), and salicylic acid (0.35% *w*/*v*). The findings were consistent with a previous study, which reported that ethanolic propolis extract exhibited significant inhibitory activity against *S. aureus* and *C. acnes*, highlighting its potential as an effective antimicrobial agent for skin-related applications [25]. Although its activity was not equivalent to that of the chemical reference compounds, propolis was noticeably more potent than honey and royal jelly. In contrast, honey and royal jelly exhibited limited or negligible antibacterial activity, particularly against *C. acnes*. Honey has been reported to exhibit broad-spectrum antimicrobial activity, likely due to its low pH, osmotic pressure, high sugar content, and the presence of bacteriostatic and bactericidal compounds [26]. However, at low concentrations, its antimicrobial efficacy may be limited, as the dose could be insufficient to fully exert these effects. Concentrations as high as 50% and 70% of Portobello and Manuka honeys have been reported to demonstrate antimicrobial activity, effectively inhibiting the majority of the bacterial species tested [27]. Moreover, royal jelly has been reported to exhibit strong antimicrobial activity against a broad spectrum of pathogens, due to the presence of bioactive compounds such as proteins, peptides, fatty acids, and phenolic compounds, which are likely to inhibit nucleic acid synthesis, reduce membrane fluidity, and suppress microbial virulence factors [28]. This suggested that while individual bee products possess some antibacterial properties, their efficacy is variable and generally lower than conventional antibiotics. Therefore, a combination of bee products, including salicylic acid, was developed to reduce the reliance on synthetic chemicals and minimize potential adverse effects [29].

Salicylic acid is a lipophilic compound that exerts comedolytic and anti-inflammatory effects [30]. Despite its therapeutic benefits, salicylic acid can cause undesirable effects, particularly with prolonged or high-concentration use [29]. Common adverse reactions include skin irritation, dryness, redness, and peeling [31]. In some individuals, salicylic acid may trigger allergic contact dermatitis or worsen existing skin sensitivity, and excessive use can lead to systemic absorption, potentially causing salicylate toxicity in rare cases [29]. For acne treatment, topical concentrations in the range of 0.5–10% were considered effective [31]. Optimized formulations containing 1.5% salicylic acid combined with natural penetration enhancers and antioxidants have demonstrated efficacy and safety for the management of mild-to-moderate acne with twice-daily application [24]. In contrast, higher concentrations of 20–30% are primarily employed as chemical peeling agents to induce exfoliation [31]. Consequently, lower concentrations are more appropriate for routine daily acne treatment, whereas higher concentrations are reserved for specialized dermatological procedures. The current study used a low concentration of salicylic acid at 0.1% *w*/*w*, which was not commonly applied topically. However, a previous report demonstrated the safe use of this concentration in glaucoma patients without ocular irritation [32]. Therefore, the selected concentration of 0.1% *w*/*w*/of salicylic acid was considered safe and unlikely to cause skin irritation. Additionally, each bee-derived component (propolis, honey, and royal jelly extract) was included at 0.25% *w*/*w*, corresponding to one-fourth of the individual test concentration (1% *w*/*w*), in order to evaluate the combined effect of all four components.

The combination of salicylic acid with propolis, honey, and royal jelly exhibited significantly greater antibacterial activity than the individual components alone. The inhibition zones measured 40.8 ± 0.8 mm for *C. acnes*, indicating a potential synergistic effect. The inhibition zones of 17.8 ± 0.3 mm for *S. aureus* and 29.0 ± 0.5 mm for *S. epidermidis* are also considered promising, especially since these effects were achieved with reduced concentrations of each component in the combination. These findings suggested that the combination of bee-derived bioactive may potentiate their antibacterial properties, particularly against acne-associated pathogens. The results highlighted the therapeutic potential of combined bee products in topical anti-acne formulations. While single bee products may provide limited efficacy, their strategic combination, potentially in conjunction with known agents like salicylic acid, can substantially enhance antibacterial effects, particularly against *C. acnes*, the primary bacterium associated with acne development. This synergistic activity underscored the potential of bee-derived formulations as complementary or alternative therapies for acne management.

### 2.2. Blank Film-Forming Gel

**Figure 1:**
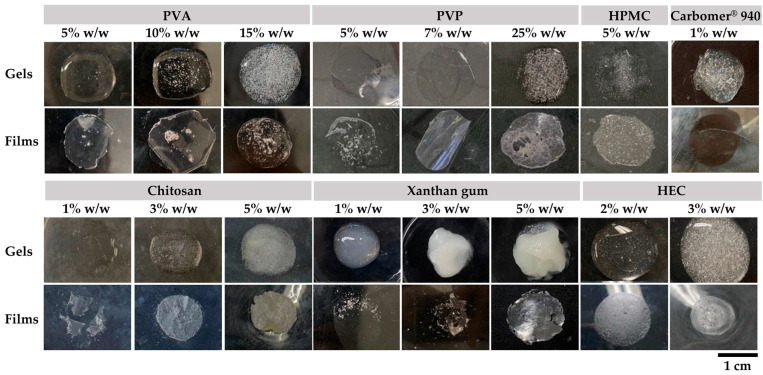
Visual appearance of gel and film formation from various polymers at different concentrations, including polyvinyl alcohol (PVA, 5–15% *w*/*w*), polyvinylpyrrolidone (PVP, 5–25% *w*/*w*), hydroxypropyl methylcellulose (HPMC, 5% *w*/*w*), Carbomer^®^ 940 (1% *w*/*w*), chitosan (1–5% *w*/*w*), xanthan gum (1–5% *w*/*w*), and hydroxyethyl cellulose (HEC, 2–3% *w*/*w*). Images represent the appearance of gels (**top row**) and their corresponding dried films (**bottom row**).

The gel-forming ability and subsequent film characteristics of different polymers at varying concentrations are presented in Figure 1, while their corresponding pH, viscosity, and drying time are summarized in Table 2.

Polyvinyl alcohol (PVA) formed transparent and homogeneous formulations. At lower concentrations (5–10% *w*/*w*), the formulations remained liquid with low viscosity (<1 mPas). Increasing the concentration to 15% *w*/*w* resulted in gel formation with markedly higher viscosity (9.2 ± 0.1 mPas), accompanied by the appearance of bubbles throughout the clear gel. This formulation was classified as a gel due to its semisolid, elastic, and deformable characteristics, which reflect the formation of a three-dimensional hydrophilic polymer network capable of absorbing and retaining substantial amounts of water [33]. The corresponding films were generally transparent. However, the higher bubble content at elevated concentrations resulted in increased surface roughness and heterogeneity of the film. The pH of all PVA gels remained constant at 5.5, regardless of the increasing concentration of the film-forming agent. PVA is valued for its biodegradability, safety, and outstanding mechanical performance [34]. In addition, it has excellent film-forming capabilities and can be easily shaped into films [34], making it suitable for use in film-forming gel formulations.

In addition, polyvinylpyrrolidone (PVP) exhibited a similar trend to PVA, forming transparent and homogeneous gels but with lower viscosity at comparable concentrations. Even at 25% *w*/*w*, the viscosity reached only 4.7 ± 0.4 mPas. The presence of bubbles was evident at higher concentrations, and the resulting films appeared more brittle than those prepared with PVA. These findings were consistent with previous research, which noted that PVP is very brittle and strongly adheres to most solid surfaces, making it difficult to remove and form a free-standing film [35]. Therefore, PVP is commonly used in combination with PVA in pharmaceutical and cosmetic formulations to produce flexible, uniform, and free-standing films [36].

On the other hand, hydroxypropyl methylcellulose (HPMC) at 5% *w*/*w* formed a highly viscous gel (23.4 ± 0.5 mPas) that was capable of film formation. Although the gel appeared transparent, the dried film became translucent with visible bubbles. Naturally, HPMC is a transparent polymer and typically forms smooth and glossy films [37]. However, the observed translucency of the film suggested that the high viscosity may have trapped air, resulting in a less uniform appearance [37]. In contrast, Carbomer^®^ 940 at 1% *w*/*w* produced a less viscous gel (12.1 ± 0.4 mPas) that appeared translucent with bubbles; however, upon drying, it yielded a transparent and homogeneous film. Carbomer^®^ 940 is a synthetic derivative of polyacrylic acid, widely used as a gelling agent due to its high viscosity and ability to form stable, transparent gels [38]. The current study highlighted its value for excellent film-forming properties, making it suitable for topical formulations in cosmetics.

Chitosan formed a transparent solution at low concentration (1% *w*/*w*); however, viscosity increased markedly with higher concentrations, leading to turbid formulations. The resulting films reflected the properties of their gels, appearing transparent at low concentrations but becoming yellowish and opaque as the polymer concentration increased. This is consistent with previous studies, which reported that chitosan films appeared yellowish and could darken further at higher drying temperatures due to Maillard browning reactions [39].

Unlike the other polymers, xanthan gum formed viscous and turbid gels at all tested concentrations (1–5% *w*/*w*). Although the gels were homogeneous with no visible bubbles, they were unsuitable for applications requiring transparency. The films produced were fragile even at high concentrations, indicating that despite the strong gel consistency, xanthan gum exhibits poor film-forming ability. The finding is consistent with the previous study. On the other hand, hydroxyethyl cellulose (HEC) formed clear and homogeneous gels at 2–3% *w*/*w*; however, at higher concentrations, the gels became very viscous and prone to bubble formation. The films obtained were opaque rather than transparent, making HEC less suitable for applications requiring clear gel films. Previous research noted that among the good film-forming cellulose ether derivatives, HEC films are perhaps the least investigated and are mainly used in combination with other polymers [40].

The drying time of the film-forming agents varied significantly depending on polymer type and concentration (*p* < 0.05). Among the tested agents, the shortest drying times were observed for xanthan gum at 5% *w*/*w* and PVP at 25% *w*/*w*. In contrast, HPMC at 5% *w*/*w* showed the longest drying time, significantly slower than all other formulations. For PVA, PVP, and xanthan gum, increasing polymer concentration led to progressively shorter drying times, suggesting that higher polymer content facilitated faster film formation and solvent evaporation. In addition, it could be attributed to the lower proportion of water in the formulation. Conversely, chitosan demonstrated the opposite trend, with drying time increasing from 22.0 ± 0.1 min at 1% *w*/*w* to 31.4 ± 0.3 min at 5% *w*/*w*, likely due to its hygroscopic nature. Previous study noted that the hydration and gelation ability of the chitosan matrix is attributed to its hygroscopic nature, which enhances its capacity to retain water [41]. Overall, these results highlighted that both the intrinsic water-binding capacity and viscosity of the polymers strongly influenced drying behavior.

Based on the comparative evaluation of various film-forming polymers, PVA in combination with Carbomer^®^ 940 was selected for further formulation development. PVA exhibited excellent film-forming ability, producing transparent and homogeneous formulations and corresponding films. However, its low viscosity resulted in liquid-like preparations. Therefore, Carbomer^®^ 940 was incorporated to enhance the overall viscosity while preserving the clarity and homogeneity of the gel, ultimately yielding transparent and uniform films. The film-forming gel formulation containing 5% *w*/*w* PVA was incorporated with various concentrations of Carbomer^®^ 940 (0.25–1% *w*/*w*), as shown in Table 3.

The incorporation of Carbomer^®^ 940 into 5% *w*/*w* PVA significantly affected the viscosity and drying time of the gel formulations and their corresponding films (*p* < 0.05), while the pH remained constant at 4.5 across all samples. The likely explanation is that the additional Carbomer^®^ 940 displaced water from the polymeric domains, thereby accelerating the drying rate [42]. At the highest concentration of Carbomer^®^ 940 (1% *w*/*w*), the formulation exhibited the greatest viscosity (17.0 ± 0.5 mPas) and the shortest drying time (14.2 ± 0.1 min). However, the gel formulation appeared slightly translucent, yielding a faintly translucent film. Reducing the concentration of Carbomer^®^ 940 resulted in lower viscosity and a longer drying time, particularly at 0.25% *w*/*w*. These findings were consistent with a previous study, which reported that the addition of Carbopol^®^ 71G significantly altered the drying time [42]. Therefore, the suggested concentration of Carbomer^®^ 940 is 0.75% *w*/*w*, as it provided high viscosity and a shorter drying time, while producing films that remain transparent and uniform.

The plasticizers, polyethylene glycol (PEG 400), glycerin, or their combination, were incorporated into the film-forming gel formulation to enhance the flexibility of the resulting films. The inclusion of these plasticizers facilitated the formation of a clear, homogeneous gel that, upon drying, produced transparent and uniform films as shown in Table 4. It was noted that both PEG 400 and glycerin significantly influenced the properties of the film-forming gels and their corresponding films. In addition, the drying time significantly increased after the addition of plasticizers, which can be attributed to the hygroscopic nature of both glycerin and PEG 400, resulting in a retardation of the drying rate [43,44]. PEG 400 alone produced films that were rigid but mechanically weak, resulting in brittleness and difficulty in removal. This behavior can be attributed to the plasticizing action of PEG 400. As a hydrophilic, low-molecular-weight compound, PEG 400 can intercalate between polymer chains, disrupting chain-to-chain hydrogen bonding and weakening intermolecular interactions within the polymeric network [45,46]. In contrast, glycerin imparted flexibility due to its strong hydrogen-bonding capacity and humectant properties [46], but at higher levels it also introduced tackiness. To overcome these limitations, a combination of PEG 400 and glycerin was employed. This approach balanced the plasticizing effect of PEG 400 with the cohesive and flexible properties provided by glycerin, thereby producing films that were smooth, flexible, and readily removable without brittleness or stickiness. The concentration of 5% *w*/*w* PEG 400 combined with 3% *w*/*w* glycerin as plasticizers produced the most suitable gel and film. A homogeneous, bubble-free gel was obtained, which formed a transparent and flexible film. Therefore, the formulation selected for further incorporation of bee-derived products consisted of 1% *w*/*w* PVA, 0.75% *w*/*w* Carbomer^®^ 940, 5% *w*/*w* PEG 400, 3% *w*/*w* glycerin, and 90.25% *w*/*w* DI water.

### 2.3. Film-Forming Gel Containing Bee Products

**Figure 2:**
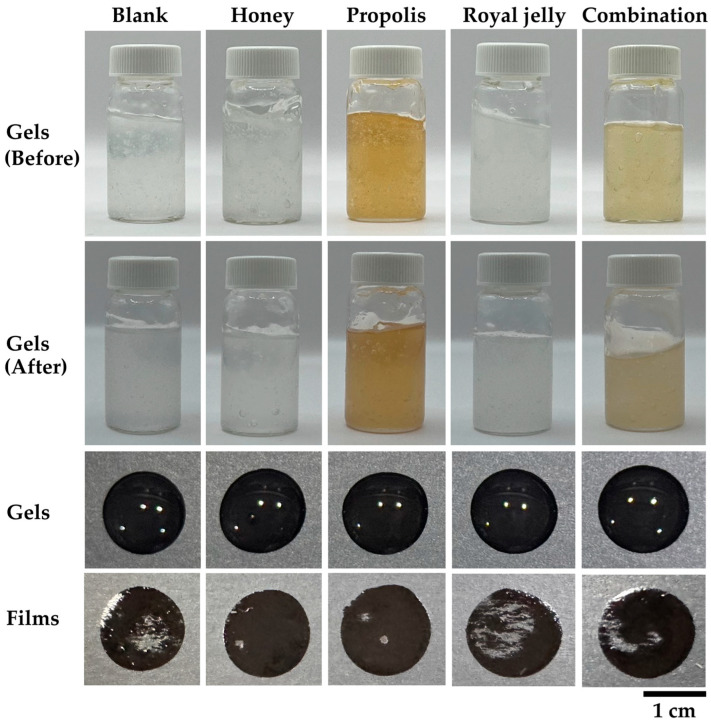
Visual appearance of film-forming gels containing 1% *w*/*w* propolis, 1% *w*/*w* honey, 1% *w*/*w* royal jelly, and their combination with 0.1% *w*/*w* salicylic acid. Top two rows show the gels before and after storage in 6 cycles of heating–cooling conditions, respectively. Third row shows gel drops on a surface, and bottom row shows the corresponding films formed from each formulation.

Bee products demonstrated notable potential in inhibiting acne-associated microorganisms, particularly when used in combination. Moreover, their mixture with salicylic acid further enhanced antibacterial activity while allowing a reduction in the amount of chemical agent required. Each bee product (propolis, honey, and royal jelly) as well as their combination with salicylic acid, was incorporated into the film-forming gel to explore their potential for anti-acne applications. The physicochemical characteristics of film-forming gels containing bee products are presented in Figure 2 and Table 5. The blank gel appeared transparent and remained stable after storage in six heating–cooling cycles. Incorporation of honey and royal jelly did not significantly alter the transparency of the gels, whereas propolis imparted a yellowish coloration. The combination of bee products produced a slightly turbid gel with a pale yellowish hue. After storage in six heating–cooling cycles, all gels maintained their homogeneity, and no phase separation was observed, indicating good stability. The findings indicated that the incorporation of honey, propolis, royal jelly, or their combination did not adversely affect gel stability, and all formulations can successfully form films. The pH values of the formulations ranged between 4.5 and 5.5, which are within the acceptable range for dermal applications and considered compatible with the skin’s natural pH (4.0–6.0) [47,48]. Among the tested formulations, the propolis extract-containing gel showed the highest pH (5.5), whereas honey and royal jelly extract maintained a lower pH of 4.5. The combination of bee products resulted in an intermediate pH (5.0), reflecting the balance among the incorporated ingredients.

In terms of viscosity, significant differences were observed following the incorporation of propolis and royal jelly extracts (*p* < 0.05), whereas honey and the combination of bee products did not exert a noticeable effect on the viscosity of the formulation. This observation could be explained by the inherent viscosity of honey, which is comparable to that of the gel matrix and therefore did not alter the overall consistency. In contrast, propolis and royal jelly extracts are less viscous, liquid-like materials, which may have interacted with the polymer network and consequently influenced the viscosity of the formulations. However, the viscosity of all gel formulations remained within a relatively narrow range, from 15.3 ± 0.2 to 16.6 ± 0.1 mPas, indicating that the variation in the formulation components did not significantly affect the properties of the gels.

Aside from the viscosity, the drying time was also affected by the incorporation of the bee products. The drying time of the gels varied significantly, ranging from 20.2 ± 1.3 to 32.3 ± 5.0 min. The royal jelly extract-containing gel demonstrated the shortest drying time, which may be explained by its relatively low viscosity and hygroscopic nature, facilitating faster solvent evaporation. Conversely, the honey-containing gel showed the longest drying time (32.3 ± 5.0 min), likely due to the high sugar content and hygroscopic property of honey, which retard water loss [49,50]. Interestingly, the combination of bee products resulted in a prolonged drying time comparable to the honey formulation, suggesting that honey plays a dominant role in determining the drying behavior of multi-component systems. However, the combination of bee products resulted in balanced characteristics, including an acceptable pH, increased viscosity, and a prolonged drying time. Since a long drying time is undesirable for film-forming gel applications, the observed drying time of approximately 30 min appears excessive. This prolonged time is likely because the measurements were conducted on a glass plate rather than human skin. Previous studies have suggested that drying time evaluated on pig skin, which better represents human skin, is considerably shorter than when tested on a glass surface [51]. Although the drying time was measured on a glass plate rather than human skin in the current study, this method was used consistently across all formulations to allow direct comparison. While the absolute drying times may be longer than those on skin, the relative differences between formulations remain valid, enabling meaningful evaluation of the effect of bee product combinations on gel performance.

### 2.4. Antimicrobial Properties of Film-Forming Gel Containing Bee Products

**Figure 3:**
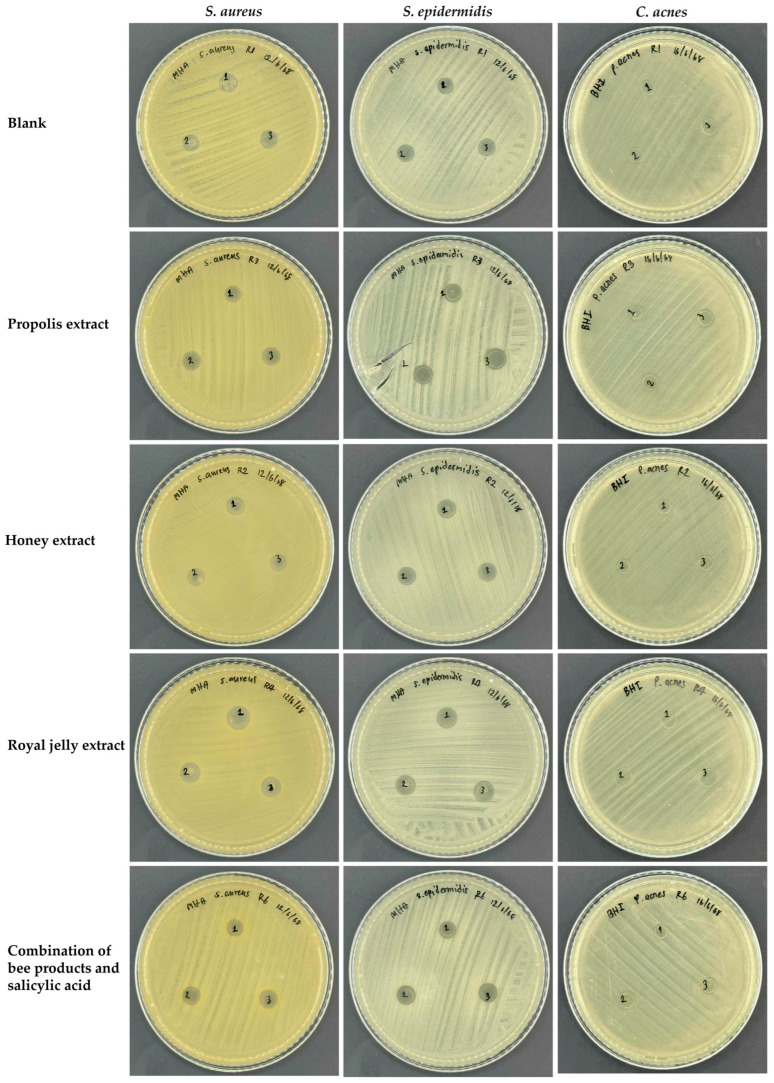
Plate inhibition drop diffusion assay demonstrating the antimicrobial activity of various film-forming gel formulations containing bee-derived products against the skin-pathogenic bacteria *S. aureus*, *S. epidermidis*, and *C. acnes*. The numbers 1, 2, and 3 in each figure indicate the results of triplicate experiments.

The antimicrobial activities of the film-forming gel formulations containing bee-derived products against skin-pathogenic bacteria are shown in Figure 3 and summarized in Table 6. The control blank film-forming gel completely inhibited the growth of *S. epidermidis*, exhibited low activity against *S. aureus*, while showing no effect on *C. acnes*. The likely explanation could be due to the physical barrier action of the polymeric ingredients rather than true antimicrobial action. PVA and Carbomer^®^ 940 were not traditional antimicrobial agents but could form very viscous matrices, preventing nutrient and oxygen access to bacteria cells and probably resulting in the bacterial growth inhibition. Both *S. epidermidis* and *S. aureus* are facultative anaerobes that can grow with or without oxygen but prefer aerobic conditions, particularly *S. epidermidis* [52], which makes it the most sensitive to the film-forming gel formulations. On the other hand, no inhibitory effect was detected for *C. acnes*, which was an anaerobe, grew under low levels of oxygen and hence not under the oxygen-limited condition of the gel base, and thus no inhibitory growth.

Incorporation of bee products had some impact on the antimicrobial properties of the film-forming gel formulations. Notably, *S. epidermidis* was highly sensitive to all bee-product-based formulations, with complete inhibition observed across treatments. However, the bee product had no additional inhibitory action, as the inhibition was complete by the blank formulation. In contrast, *C. acnes* demonstrated resistance to all formulations, maintaining high bacterial growth irrespective of the incorporated bee product. Interestingly, the combination of salicylic acid and bee products totally inhibited the growth of *S. aureus*. Among individual bee products, 1% *w*/*v* propolis extract exhibited the least inhibitory towards *S. aureus*, while 1% *w*/*v* honey and 1% *w*/*v* royal jelly extract exhibited high inhibitory effect. As their combination totally inhibited *S. aureus*, these results remarked that synergistic inclusion broadened the antibacterial spectrum, which indicated the potential of such anti-acne mixtures to produce effective film-forming gels. However, further clinical studies would be recommended, as the in vitro results may be limited by factors such as solubility and formulation characteristics.

## 3. Conclusions

This study highlighted the significant potential of bee-derived products, both individually and in combination with low-dose salicylic acid, as effective antibacterial agents in topical film-forming gels for acne management. Among the tested bee products, propolis (1% *w*/*w*) demonstrated the most potent activity, producing an inhibition zone of 20.0 ± 1.0 mm against *S. aureus*. Notably, the combination of bee products with salicylic acid significantly enhanced antibacterial efficacy, particularly against *C. acnes* (inhibition zone 40.8 ± 0.8 mm). Therefore, this combination was incorporated into the film-forming gel formulation, with the most suitable formulation containing 1% *w*/*w* PVA, 0.75% *w*/*w* Carbomer^®^ 940, 5% *w*/*w* PEG 400, 3% *w*/*w* glycerin, and the remainder as water to achieve the desired gel consistency and optimal film-forming properties. After incorporating the bee product combination (0.1% *w*/*w* salicylic acid, 0.25% *w*/*w* propolis extract, 0.25% *w*/*w* honey, and 0.25% *w*/*w* royal jelly extract), the formulation achieved a balanced pH (5.0), optimal viscosity (16.6 ± 0.1 mPas), and a drying time of 31 min. Although drying times measured on glass plates were longer than expected for human skin, consistent methodology allowed reliable comparison. The film-forming gel containing a low dose of salicylic acid (0.1% *w*/*w*) in combination with bee-derived products completely inhibited the growth of *S. aureus* and *S. epidermidis*. Therefore, these findings supported the feasibility of multi-component bee product gels as complementary or alternative anti-acne therapies, reducing dependence on conventional chemicals while retaining efficacy and skin compatibility. The novelty of this research lies in both the formulation design and the unique active combination. However, additional physicochemical characterizations, including Fourier-transform infrared spectroscopy, nuclear magnetic resonance spectroscopy, X-ray diffraction, and differential scanning calorimetry, would be recommended to provide deeper insight into the gel structure and the formation of polymeric network. On the other hand, further in vivo studies are required to confirm clinical applicability.

## 4. Materials and Methods

### 4.1. Bee Products and Chemical Materials

Salicylic acid (35% *w*/*v* in isopentyldiol), propolis extract (>0.5% *w*/*w* flavonoid content in glycerin), and royal jelly extract (3.8–4.2% *w*/*w* 10-hydroxydecanoic acid in the formulation containing polyethylene glycol-40 hydrogenated castor oil, ethoxydiglycol, tocopheryl acetate, and water) were cosmetic grade purchased from Chanjao Longevity Co., Ltd. (Bangkok, Thailand). Honey was purchased from local market in Chiang Mai, Thailand. PVA (PVA 205; (C_2_H_3_OR)_n_; MW 44.05 g/mol; degree of saponification ~87.0 to ~89.0 mol%; degree of polymerization ~500), PVP (PVP-K15; (C_6_H_9_NO)_n_; MW 111.14 g/mol g/mol), HPMC (C_56_H_108_O_30_; MW 1261.42 g/mol), HEC (C3H7O^•^; MW 59.09 g/mol), Carbomer^®^ 940 (Sigma-Aldrich, St. Louis, MO, USA) (Carbopol 940; (C_3_H_4_O_2_)_n_; MW 72.06 g/mol), chitosan (C_6_H_11_NO_4×2_; MW 161.16 + (2X) g/mol), xanthan gum (C_35_H_49_O_29_; MW 933.74 g/mol), triethanolamine (C_6_H_15_NO_3_; MW 149.19 g/mol), PEG 400 (H(OCH_2_CH_2_)_n_OH; MW 400 g/mol), and glycerin (C_3_H_8_O; MW 92.09 g/mol) were analytical grade purchased from Sigma-Aldrich (St. Louis, MO, USA). Mueller–Hinton Broth (MHB), Mueller–Hinton Agar (MHA), as well as Brain Heart Infusion (BHI) broth and agar media were obtained from HiMedia (Dindori, Nashik, India). Dimethyl sulfoxide (DMSO) and sodium chloride (NaCl) were obtained from RCI Labscan (Pathumwan, Bangkok, Thailand). Gentamicin and tetracycline were purchased from BIO BASIC Inc. (Markham, ON, Canada).

### 4.2. Antimicrobial Test of Bee Products

The antimicrobial activity of each bee product sample and their combinations was evaluated using the agar-well diffusion method [53]. Three skin pathogenic bacteria, namely *S. aureus* DMST8013, *S. epidermidis* DMST5868, and *C. acnes* DMST14916, were used in this study. Both *S. aureus* and *S. epidermidis* were cultured in MHB and incubated at 37 °C for 24 h under aerobic conditions, while *C. acnes* was cultured in BHI broth at 37 °C for 72 h under anaerobic conditions. The bacterial suspensions were adjusted to a turbidity equivalent to the 0.5 McFarland standard (approximately 1 × 10^8^ CFU/mL) using 0.85% NaCl. Suspensions of *S. aureus* and *S. epidermidis* were swabbed onto MHA plates, whereas those of *C. acnes* were swabbed onto BHI agar plates using sterile cotton swabs. Plates were punched with a sterile 6 mm diameter Cork Borer, and 50 µL of the test samples were added to each well. For controls, 100% DMSO was used as a negative control for samples dissolved in DMSO, while gentamicin (0.1 mg/mL) and tetracycline (0.1 mg/mL) served as positive controls. Plates inoculated with *S. aureus* and *S. epidermidis* were incubated aerobically at 37 °C for 24 h, whereas those with *C. acnes* were incubated anaerobically at 37 °C for 72 h. The antibacterial activity was determined by measuring the diameters of the inhibition zones (in millimeters) using a vernier caliper. All experiments were performed in triplicate.

### 4.3. Development of Blank Film-Forming Gel

#### 4.3.1. Preparation of Blank Film Forming Gel

The effects of polymer and plasticizer type and concentration on the properties of film-forming gels were evaluated. Film-forming gels were prepared using various polymers, including PVA, PVP, HPMC, HEC, Carbomer^®^ 940, chitosan, xanthan gum, and a PVA/Carbomer^®^ 940 combination, in order to identify suitable film-forming bases. Each polymer was dispersed in DI water under continuous stirring until completely hydrated. For PVA, the solution was heated to 70–80 °C until fully dissolved. Carbomer^®^ 940 gels were neutralized to pH 6.0–7.0 using triethanolamine, while chitosan was dissolved in acetate buffer pH 3.0 under magnetic stirring (IKA^®^ C-MAG HS7, IKA Werke GmbH & Co. KG, Staufen, Germany). Based on the initial screening, the most promising polymers were selected for further optimization with plasticizers. PEG-400 and glycerol were incorporated at concentrations of 3–10% *w*/*w*, followed by homogenization to ensure uniform distribution. The resulting formulations were subjected to physicochemical characterization and film property assessments to determine the optimal polymer–plasticizer combinations.

#### 4.3.2. Characterization of Physical Characterization of Blank Film-Forming Gels

The physical properties of the film-forming gel formulations were evaluated through visual observation, pH determination, and viscosity measurement. The appearance of each formulation was examined for clarity, homogeneity, and phase separation by direct visual inspection. The pH of the gels was measured using a calibrated digital pH meter (Inolab Level 2, WTW/Inolab, Weilheim, Germany) at room temperature. Viscosity was determined using a cone-plate measuring system (R/S Rheometer, Brookfield Viscometer LTD., Middleboro, United States) set at 25 °C [54]. Each measurement was performed in triplicate. To evaluate the physical stability of the film-forming gel formulations, each formulation was stored in well-closed containers under six heating–cooling cycles, alternating between 45 °C for 24 h and 4 °C for 24 h. Afterward, the formulations were evaluated by visual observation.

#### 4.3.3. Evaluation of Film Properties of Blank Film-Forming Gels

Physical appearance and drying time of blank film-forming gels were assessed. The physical appearance of each film formed from the gel formulations was first assessed by visual inspection for clarity, color, and homogeneity. Drying time, defined as the period required for the film-forming gel base to completely dry, was assessed using the glass plate method [51]. In brief, 50 mg of each gel formulation was applied to a glass slide to cover an area of 1.8 × 1.8 cm. Immediately after application, a timer was started. The evaporation process was monitored by visual observation, and the weight of the glass slide with the applied formulation was measured periodically. Drying time was recorded as the point at which the weight remained constant, indicating complete drying of the gel. Each formulation was evaluated in triplicate, and the mean drying time was calculated and reported.

### 4.4. Development of Film-Forming Gel Containing Bee Products

The most suitable film-forming gel formulation, consisting of 1% *w*/*w* PVA, 0.75% *w*/*w* Carbomer^®^ 940, 5% *w*/*w* PEG 400, 3% *w*/*w* glycerin, and 90.25% *w*/*w* DI water, was selected based on its physical properties and film-forming performance. Each bee product (honey extract, propolis extract, and royal jelly) and their combination, at a concentration of 1% *w*/*w* with 0.1% salicylic acid, was incorporated into the most suitable film-forming gel. In brief, PVA and Carbomer^®^ 940 were separately dispersed in DI water under continuous stirring until homogeneous gels were obtained. In parallel, honey extract, propolis extract, royal jelly, and salicylic acid were dissolved in a mixture of PEG 400 and glycerin with DI water. The resulting solution was gradually incorporated into the preformulated gels. The resulting formulations were thoroughly mixed to ensure uniform distribution of the active ingredients, followed by characterization of their physicochemical properties, drying time, and film properties as described above.

### 4.5. Antimicrobial Test of Film-Forming Gel Containing Bee Products

The antibacterial activity of the film-forming gel containing bee products formulation was evaluated using the drop diffusion assay. The bacterial inoculum was prepared same as in Section 4.2. The bacterial suspension was swabbed on MHA and BHI agar plates using sterile cotton swabs. Subsequently, 50 µL of the testing sample was carefully dropped onto the surface of the plates, ensuring that the droplets did not touch each other. The samples were allowed to diffuse for 15–20 min following the method described by Rauwel et al. (2024) [55]. Plates inoculated with *S. aureus* and *S. epidermidis* were incubated aerobically at 37 °C for 24 h, while those inoculated with *C. acnes* were incubated anaerobically at 37 °C for 72 h. After incubation, the antibacterial effect was assessed by observing the bacterial growth beneath and around the drop area. All experiments were performed in triplicate. The antibacterial activity was classified into four categories: no inhibitory activity or high bacterial growth (++++ and +++), low activity (++), high activity (+), and complete inhibition or no detectable bacterial growth (−), as proposed by Sutjarittangtham et al. (2014) [56].

### 4.6. Statistical Analysis

All data were expressed as mean ± standard deviation (SD). Statistical analysis was performed using one-way analysis of variance (ANOVA) followed by post hoc comparisons in SPSS version 17.0 for Windows (SPSS Inc., Chicago, IL, USA). Differences were considered statistically significant at * *p* < 0.05, ** *p* < 0.01, and *** *p* < 0.001.

## Figures and Tables

**Table 1:** Antibacterial effects of bee products against acne-related bacteria.

Samples	Inhibition Zone (mm)		
	*S. aureus*	*S. epidermidis*	*C. acnes*
Gentamicin (0.1 mg/mL)	24.3 ± 2.3 ^a^	23.5 ± 0.5 ^c^	29.7 ± 2.5 ^b^
Tetracycline (0.1 mg/mL)	29.0 ± 2.2 ^a^	33.2 ± 0.8 ^a^	42.7 ± 1.2 ^a^
DMSO (100%)	0.0 ± 0.0 ^d^	0.0 ± 0.0 ^f^	0.0 ± 0.0 ^d^
Salicylic acid (0.35% *w*/*v*)	27.3 ± 0.8 ^a^	30.2 ± 0.8 ^b^	43.0 ± 1.0 ^a^
Propolis extract (1% *w*/*v*)	20.0 ± 1.0 ^a,b^	24.8 ± 0.3 ^c^	23.0 ± 1.0 ^c^
Honey (1% *w*/*v*)	10.8 ± 0.3 ^c^	14.2 ± 0.3 ^d^	0.0 ± 0.0 ^d^
Royal jelly extract (1% *w*/*v*)	8.3 ± 3.6 ^c^	10.3 ± 0.8 ^e^	0.0 ± 0.0 ^d^
Combination of bee products	17.8 ± 0.3 ^b^	29.0 ± 0.5 ^b^	40.8 ± 0.8 ^a^

Note: DMSO = dimethyl sulfoxide, which was used as a vehicle control; combination of bee products containing 0.1% *w*/*w* salicylic acid, 0.25% *w*/*w* propolis extract, 0.25% *w*/*w* honey, and 0.25% *w*/*w* royal jelly extract. Different letters (a–f) indicate significant differences between samples (*p* < 0.05).

**Table 2:** Characteristics of film-forming gels.

Film-Forming Agent	Concentration (% *w*/*w*)	pH	Viscosity (mPas)	Drying Time (min)
PVA	5	5.5	<1.0 ^i^	35.2 ± 0.2 ^j^
	10	5.5	<1.0 ^i^	25.0 ± 0.1 ^g^
	15	5.5	9.2 ± 0.1 ^d^	23.0 ± 0.1 ^e^
PVP	5	5.0	<1.0 ^i^	22.0 ± 0.1 ^d^
	7	5.0	<1.0 ^i^	20.1 ± 0.1 ^c^
	25	5.0	4.7 ± 0.4 ^f^	18.5 ± 0.1 ^a^
HPMC	5	5.5	23.4 ± 0.5 ^a^	38.5 ± 0.1 ^k^
Carbomer^®^ 940	1	5.0	12.1 ± 0.4 ^c^	20.4 ± 0.1 ^c^
Chitosan	1	4.0	<1.0 ^i^	22.0 ± 0.1 ^d^
	3	5.0	7.7 ± 0.2 ^e^	28.3 ± 0.2 ^i^
	5	6.0	14.7 ± 0.9 ^b^	31.4 ± 0.3
Xanthan gum	1	5.0	2.1 ± 0.1 ^h^	25.5 ± 0.2 ^g^
	3	5.0	9.5 ± 0.3 ^d^	19.4 ± 0.1 ^b^
	5	5.0	15.4 ± 0.2 ^b^	18.1 ± 0.1 ^a^
HEC	2	5.5	3.5 ± 0.3 ^g^	26.4 ± 0.1 ^h^
	3	5.5	9.6 ± 0.1 ^d^	24.3 ± 0.1 ^f^

Note: PVA = polyvinyl alcohol; PVP = polyvinylpyrrolidone; HPMC = hydroxypropyl methylcellulose; HEC = hydroxyethyl cellulose. Different letters (a–k) indicate significant differences between samples (*p* < 0.05).

**Table 3:** Characteristics of film-forming gels containing a combination of PVA and Carbopol^®^ 940 and their corresponding films.

Concentration (% *w*/*w*)		Visual Appearance		pH	Viscosity (mPas)	Drying Time (min)
PVA	Carbomer^®^ 940	Gel	Film			
5	1	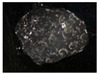	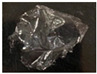	4.5	17.0 ± 0.5 ^a^	14.2 ± 0.1 ^a^
5	0.75	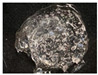	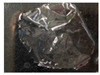	4.5	14.8 ± 0.8 ^b^	16.4 ± 0.1 ^b^
5	0.50	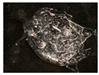	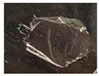	4.5	10.7 ± 0.7 ^c^	16.3 ± 0.1 ^b^
5	0.25	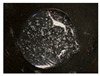	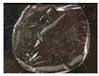	4.5	3.3 ± 0.3 ^d^	21.3 ± 0.1 ^c^

Note: PVA = polyvinyl alcohol; Different letters (a–d) indicate significant differences between samples (*p* < 0.05).

**Table 4:** Characteristics of film-forming gels containing a combination of PVA and Carbopol^®^ 940, with PEG 400 or glycerin as plasticizers, and their corresponding films.

Concentration (% *w*/*w*)				Visual Appearance		pH	Viscosity (mPas)	Drying Time (min)
PVA	Carbomer^®^ 940	PEG 400	Glycerin	Gel	Film			
5	0.75	-	-	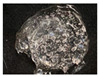	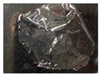	4.5	14.8 ± 0.8 ^e^	16.4 ± 0.1 ^g^
5	0.75	10	-	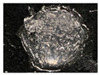	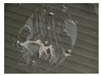	4.5	14.6 ± 0.1 ^e^	31.3 ± 0.1 ^e^
5	0.75	5	-	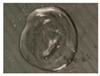	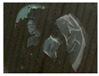	4.5	15.6 ± 0.1 ^d^	32.1 ± 0.1 ^d^
5	0.75	5	3	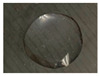	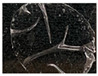	4.5	16.3 ± 0.1 ^c^	27.4 ± 0.1 ^f^
5	0.75	-	3	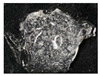	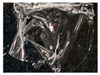	4.5	17.3 ± 0.3 ^b^	39.3 ± 0.1 ^a^
5	0.75	-	5	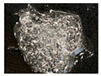	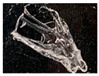	4.5	17.4 ± 0.3 ^b^	37.2 ± 0.1 ^b^
5	0.75	-	10	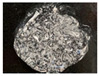	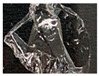	4.5	17.0 ± 0.2 ^b^	33.2 ± 0.1 ^c^

Note: PVA = polyvinyl alcohol; PEG 400 = polyethylene glycol 400; Different letters (a–g) indicate significant differences between samples (*p* < 0.05).

**Table 5:** Characteristics of film-forming gels containing bee products.

Film-Forming Gel Formulation	pH	Viscosity (mPas)	Drying Time (min)
Blank	4.5	16.3 ± 0.1 ^a^	27.4 ± 0.1 ^b^
Propolis extract (1% *w*/*v*)	5.5	15.3 ± 0.2 ^c^	25.2 ± 2.2 ^b^
Honey (1% *w*/*v*)	4.5	15.9 ± 0.4 ^a,b^	32.3 ± 5.0 ^a^
Royal jelly extract (1% *w*/*v*)	4.5	15.6 ± 0.1 ^b,c^	20.2 ± 1.3 ^c^
Combination of bee products and salicylic acid	5.0	16.6 ± 0.1 ^a^	31.3 ± 1.2 ^a^

Note: A combination of bee products containing 0.1% *w*/*w* salicylic acid, 0.25% *w*/*w* propolis extract, 0.25% *w*/*w* honey, and 0.25% *w*/*w* royal jelly extract. Different letters (a–c) indicate significant differences between samples (*p* < 0.05).

**Table 6:** Antibacterial activity of film-forming gel formulations containing bee products.

Film-Forming Gel Formulation	Bacterial Growth		
	*S. aureus*	*S. epidermidis*	*C. acne*
Blank	++	−	++++
Propolis extract (1% *w*/*v*)	+++	−	++++
Honey (1% *w*/*v*)	+	−	++++
Royal jelly extract (1% *w*/*v*)	+	+	++++
Combination of bee products and salicylic acid	−	−	++++

Note: A combination of bee products containing 0.1% *w*/*w* salicylic acid, 0.25% *w*/*w* propolis extract, 0.25% *w*/*w* honey, and 0.25% *w*/*w* royal jelly extract. No inhibitory activity or high bacterial growth (++++ and +++), low activity (++), high activity (+), and complete inhibition or no detectable bacterial growth (−).

## Data Availability

All relevant data are included within the article.

## Abbreviations

The following abbreviations are used in this manuscript:

ANOVA	analysis of variance
BHI	Brain heart infusion
DMSO	Dimethyl sulfoxide
HEC	Hydroxyethyl cellulose
HPMC	Hydroxypropyl methylcellulose
MHA	Mueller–Hinton agar
MHB	Mueller–Hinton broth
PEG 400	Polyethylene glycol 400
PVA	Polyvinyl alcohol
PVP	Polyvinylpyrrolidone
SD	standard deviation
